# Yiqi Jianpi Huayu Jiedu Decoction Inhibits Metastasis of Colon Adenocarcinoma by Reversing Hsa-miR-374a-3p/Wnt3/β-Catenin-Mediated Epithelial–Mesenchymal Transition and Cellular Plasticity

**DOI:** 10.3389/fonc.2022.904911

**Published:** 2022-06-28

**Authors:** Yuwen Zhuang, Jinyong Zhou, Shenlin Liu, Qiong Wang, Jun Qian, Xi Zou, Haiyan Peng, Tian Xue, Zhichao Jin, Cunen Wu

**Affiliations:** ^1^ Department of Oncology, Affiliated Hospital of Nanjing University of Chinese Medicine, Jiangsu Province Hospital of Chinese Medicine, Nanjing, China; ^2^ Central Laboratory, Affiliated Hospital of Nanjing University of Chinese Medicine, Jiangsu Province Hospital of Chinese Medicine, Nanjing, China; ^3^ Department of Education, Affiliated Hospital of Nanjing University of Chinese Medicine, Jiangsu Province Hospital of Chinese Medicine, Nanjing, China

**Keywords:** Yiqi Jianpi Huayu Jiedu decoction, colon adenocarcinoma, epithelial–mesenchymal transition, cellular plasticity, hsa-miR-374a-3p, Wnt3

## Abstract

Colon adenocarcinoma (COAD) accounts for 95% of colon cancer cases, with the 5-year survival rate significantly affected by local or distant metastases. Yiqi Jianpi Huayu Jiedu decoction (YJHJD), based on the theory of “nourish qi, invigorate the spleen, remove blood stasis, and detoxify”, has long been applied and shown to be remarkable in the prevention and treatment of gastrointestinal tumors. However, the underlying therapeutic mechanisms of YJHJD have not been fully elucidated. Herein, we first confirmed hsa-miR-374a-3p as a tumor suppressor based on its lower expression in the plasma of patients with COAD with liver metastasis and association with more advanced local progression. We also verified WNT3 as a potential target of hsa-miR-374a-3p and observed its increased expression in COAD tissues. Furthermore, we showed that the hsa-miR-374a-3p/Wnt3/β-catenin axis was responsible for epithelial–mesenchymal transition (EMT) and cellular plasticity in COAD, as well as poorer patient prognosis. Our results showed that YJHJD inhibited motility and colony potential *in vitro*, as well as liver metastasis of COAD *in vivo*. Moreover, YJHJD induced a reversal of EMT and cellular plasticity-related molecular expression, increased hsa-miR-374a-3p, and decreased Wnt3 and β-catenin levels. In addition, silencing of hsa-miR-374a-3p weakened YJHJD inhibition, whereas the β-catenin inhibitor XAV939 partially repaired it. Taken together, these results demonstrated that YJHJD suppressed the EMT and cellular plasticity of COAD by regulating hsa-miR-374a-3p/Wnt3/β-catenin signaling.

## Introduction

Colon cancer is the fifth most common malignancy worldwide, causing nearly 1,150,000 new cases and 580,000 deaths annually. Colon adenocarcinoma (COAD) is one of the most common pathological subtypes, accounting for 95% of colon cancer cases (1). With increased control of high-risk factors and improvements in diagnosis and treatment techniques, the incidence of colon cancer has been declining in recent years; however, more than half of patients have local or distant metastases when diagnosed, severely affecting patient 5-year survival (2, 3). Although intravenous chemotherapy, targeted therapy, radiotherapy, and immunotherapy can control lesions to a certain extent and benefit patients, treatment efficiency is often limited by drug resistance or side effects (4). Therefore, reducing the risk of metastasis is an effective strategy for improving the comprehensive efficacy and prognosis of patients with COAD.

The epithelial–mesenchymal transition (EMT) is an important process by which epithelial cells acquire mesenchymal properties. This process is related to a variety of biological characteristics of tumors, including metastasis. During the EMT, the epithelial molecule E-cadherin is downregulated, and the mesenchymal marker N-cadherin and a series of EMT-activating transcription factors such as Snail are upregulated. Tumor cells lose their original polarity and close intercellular connections, thus acquiring stronger infiltrative and invasive abilities during EMT (5). Furthermore, cancer cell plasticity is necessary for EMT during metastasis (6). EMT-induced cellular plasticity and cancer stem cell (CSC)-related signals are associated with malignant progression (7). CSCs are a small group of tumor-initiating cells (TICs) that can produce multiple cell types through self-renewal and differentiation, subsequently leading to tumor initiation. CSCs are closely related to COAD metastasis due to their insensitivity to traditional treatment methods (8). Reversing the EMT and cellular plasticity to reduce metastatic potential may be an effective method for the treatment of COAD metastasis.

Increasing evidence has shown that microRNAs (miRNAs) are involved in multiple processes, including malignancy genesis and development. By specifically binding to the 3’-UTR region of the target mRNA, miRNAs can regulate mRNA translation or degradation and play a role in carcinogenesis or tumor inhibition (9). Among these miRNAs, hsa-miR-374a was shown to participate in the formation of a negative feedback loop and suppression of downstream signals associated with EMT in nasopharyngeal carcinoma (10). Moreover, hsa-miR-374a suppressed *in vivo* tumorigenicity by reducing the stemness of human glioma stem cells (11). Hsa-miR-374a also directly targeted regulators of the Wnt/β-catenin pathway to regulate EMT in breast cancer (12). Wnt3a can induce β-catenin accumulation and RhoA activation, which is linked to upregulated vimentin expression (13). Therefore, targeted regulation of the hsa-miR-374a-3p/Wnt3 axis may provide a new direction for the prevention and treatment of COAD metastasis.

Traditional Chinese medicine theory states that “spleen deficiency and stasis toxin” are the main pathogenesis of COAD, while the combination of “qi deficiency,” “blood stasis”, and “cancer toxin” is a key pathological factor. Hence, the treatment principle follows the theory of “nourish qi, invigorate the spleen, remove blood stasis, and detoxify”. Based on this, a traditional Chinese medicine compound prescription derived from the classical formula Guishao Liujun decoction, called Yiqi Jianpi Huayu Jiedu decoction (YJHJD), has long been applied for the treatment of COAD. To further explore the mechanism underlying the inhibitory effect of YJHJD on COAD, we investigated the influence of YJHJD on the EMT and cellular plasticity of COAD cells, as well as YJHJD regulation of hsa-miR-374a-3p/Wnt3 signaling.

## Materials and Methods

### RNA Extraction and Small RNA Sequencing Analysis

Peripheral venous blood was collected from each subject early in the morning on an empty stomach. Plasma was isolated within 4 h and stored at −80°C for future analysis. Total cell-free RNA was extracted from the plasma using the miRNeasy Serum/Plasma kit (Qiagen, CA, USA). A small RNA library was established using a TruSeq Small RNA Sample Prep Kit (Illumina, San Diego, CA, USA). The qualified library was loaded onto an Illumina HiSeq 2500 platform for high-throughput sequencing (Lianchuan Biotechnology, Hangzhou, China). Raw data from each sample were filtered using ACGT101-miR (LC Sciences, Houston, Texas, USA) to remove junk sequences, adapter dimers, low-complexity reads, common RNA families, and repeats. Known miRNAs and novel 5p or 3p miRNAs were identified by mapping all 18–26 nucleotide sequences to the miRBase22.0 database (http://www.mirbase.org/). The remaining sequences were aligned to the miRBase miRNA database to identify completely matched sequences that were judged to be conserved *Homo sapiens* miRNAs (14). Differences in miRNA expression were evaluated by analysis of variance (ANOVA) test, with the significance threshold defined as |log2 FC| > 1.0 and *p* < 0.05.

### Plasma and Tissue Samples of Patients With COAD

A total of 66 patients diagnosed with primary COAD by postoperative pathology were recruited for this retrospective study. All plasma specimens were collected by centrifuging peripheral venous blood preoperatively and stored at −80°C. Another three pairs of para-carcinoma, CAOD, and hepatic metastasis tissue samples were collected from three patients who were diagnosed with COAD with hepatic metastasis immediately after surgical resection and frozen in liquid nitrogen within 5 min. Written informed consent was obtained from all the patients.

### Data Source and Different Expression Gene Analysis

COAD data containing 41 paracancerous and 480 COAD samples were acquired from The Cancer Genome Atlas (TCGA) database. Annotation information (GENCODE v25) was obtained from the UCSC Xena database. Differentially expressed genes (DEGs) between paracancerous tissues and tumors were authenticated *via* the classical Bayesian method in the Limma package (Version 3.10.3), with a threshold value of adjusted *p* < 0.01 and |log2 FC| > 1.5. Heatmaps and volcano figures of DEGs were constructed using the pheatmap and ggplot packages.

### Kaplan–Meier Plotter Analysis

The patients with COAD were divided into low and high groups according to the specific miRNA or mRNA expression levels. The relationships between miRNA or mRNA expression and the prognosis of patients with COAD were assessed based on the Kaplan–Meier method using the survival package and visualized using the survminer package. Statistical significance was defined as *p* < 0.05.

### Cell Culture

The human COAD cell lines HCT116 and HT29 were obtained from the Type Culture Collection, Chinese Academy of Sciences (Shanghai, China). The mouse colon cancer cell line CT26-GFP was purchased from Cellcook (Guangzhou, China). The cells were cultured in RPMI-1640 medium with 10% fetal bovine serum (FBS, Gibco-BRL, Gaithersburg, USA) in a humidified incubator at 37°C and 5% CO_2_.

### Fluorescence *In Situ* Hybridization and Immunofluorescence in Human Tissues

FISH experiments were conducted to evaluate hsa-miR-374a-3p expression in human tissues as described previously (15, 16). Hsa-miR-374a-3p probes [5’-AATTACAATACAATCTGATAAG (ttt CATCATCAT ACATCATCAT)30-3’, 5’-DIG-tt ATGATGATGT ATGATGATGT-3’] with a double digoxigenin (DIG)-labeled probe against hsa-miR-374a-3p were synthesized (Servicebio Technology, Wuhan, China). For *in situ* hybridization, anti-DIG-horseradish peroxidase (HRP)-conjugated antibody (Jackson, West Grove, USA) and tyramide signal amplification iF647-TSA (Servicebio) were applied and the nuclei were stained with 4′,6-diamidino-2-phenylindole (DAPI). Wnt3 (1:100, Abcam, Cambridge, UK), β-catenin (1:100, Servicebio, Wuhan, China), and Cy3/FITC-labeled anti-rabbit immunoglobulin G (IgG, Servicebio, Wuhan, China) were used for the analysis of human specimens. Fluorescence images were captured using a Nikon imaging system (DS-U3; Tokyo, Japan).

### Transfection of COAD Cells With Hsa-miR-374a-3p Mimic/Inhibitor

Hsa-miR-374a-3p mimic/inhibitor and scrambled control plasmids were constructed by Gene Pharma (Shanghai, China). HCT116 and HT29 cells were seeded in 24-well plates and incubated for 24 h. Both cell types were then transfected with miRNA-335-5p mimic/inhibitor using Lipofectamine 3000 Transfection Reagent according to the manufacturer’s protocol (Invitrogen, Grand Island, NY, USA). Transfection potency was confirmed 48 h later by RT-PCR.

### Wound-Healing Assay

HCT116 and HT29 cells were plated in six-well dishes and incubated overnight to form confluent monolayers. Pipette tips were used to scratch a strip to create a line across the cell monolayers. The remaining cells underwent another 24-h incubation and the relative wounded areas were measured and calculated.

### Transwell Assay

Transwell membrane filter inserts (pore size, 8 μm; Costar, Corning, NY, USA) in 24-well dishes were used to test the cell invasive ability. Starved COAD cells were resuspended in serum-free medium (5 × 10^5^/ml). A 200-μl cell suspension was added to the upper inserts and covered with Matrigel. Then, 500 μl of complete medium was added to the lower chambers. After another 24-h incubation, the cells that remained on the upper side were wiped off. The cells were fixed with 95% alcohol and stained with 0.05% crystal violet for further analysis.

### Spheroid Formation

HCT116 and HT29 cells (1×10^3^/well) were seeded into ultralow attachment six-well plates, filled with serum-free medium containing 1% B27 (Life Technology, Grand Island, USA), 20 ng/ml epidermal growth factor (EGF), and 10 ng/ml basic fibroblast growth factor (bFGF, Peprotech, Rocky Hill, USA) at 37°C in 5% CO_2_. The medium was changed every 6 days. Spheroid formation images were obtained 10 or 20 days after culture.

### RT-PCR Assay

Total RNA was extracted from COAD cells or liver metastatic tumor tissues using TRIzol reagent (Life Technology, Grand Island, USA) and reverse-transcribed into cDNA using the PrimeScript RT reagent kit with gDNA Eraser (TaKaRa, Dalian, China). The primers used in this experiment are presented in **Table 1**. Gene expression was investigated on an ABI7500 fast RT-PCR System (Applied Biosystems, Waltham, USA) using the DNA-binding SYBR-Green dye and the △△Ct analysis method.

**Table 1:** Primers used for RT-PCR.

Target	Primer sequence
hsa-miR-374a-3p	Forward 5’-TACATCGGCCATTATAATA-3’
	Reverse 5’-TACACAGAATTACAATAC-3’
U6	Forward 5’-CTCGCTTCGGCAGCACA-3’
	Reverse 5’-AACGCTTCACGAATTTGCGT-3’
WNT3	Forward 5’-CGTCTTCCACTGGTGCTGC-3’
	Reverse 5’-CAGTCCATGCTCCTTGCTG-3’
GAPDH	Forward 5’-TGGGTGTGACCATGAGAAGT-3’
	Reverse 5’-TGAGTCCTTCCACGATACCAA-3’

### Western Blot Assay

Total or nuclear proteins were extracted from COAD cells or liver metastatic tumor tissues and lysed in radioimmunoprecipitation assay (RIPA) buffer. Proteins were separated by sodium dodecyl sulfate-polyacrylamide gel electrophoresis (SDS-PAGE) and transferred to polyvinylidene difluoride (PVDF) membranes (Millipore, Bedford, USA). Membranes were incubated with 5% bovine serum albumin (BSA) for 1 h at room temperature and then incubated with primary antibodies against E-cadherin, N-cadherin, Snail, CD44, Sox2, β-catenin (1:1,000, CST), β-actin, histone H3 (1:2,000, CST), and Wnt3 (1:1,000, Abcam) at 4°C overnight. The membranes were then incubated with secondary antibodies at room temperature for 1 h. Protein signals were probed using an ECL kit (BI, Kibbutz Beit-Haemek, Israel).

### Immunofluorescence Analysis

The cells were fixed with 4% paraformaldehyde for 15 min and permeabilized with 0.5% Triton X-100 for 20 min. Subsequently, 5% BSA was used to block the cells for 30 min. The cells were incubated with antibodies against Wnt3 (1:100, Abcam, Cambridge, UK) or β-catenin (1:100; Servicebio, Wuhan, China) overnight at 4°C followed by incubation with Cy3-labeled anti-rabbit IgG (1:200 dilution, Servicebio, Wuhan, China) secondary antibody for 1 h. Next, the nuclei were stained with DAPI for 5 min. Fluorescence images were acquired using a laser scanning confocal microscope (Zeiss LSM710, Jena, Germany).

### Immunohistochemistry Analysis

Formaldehyde-fixed portions of the tissues were embedded in paraffin and sectioned into 4-μm thick slices. After deparaffinization in xylene, the sections were hydrated in a series of graded alcohols. A pressure cooker was used to perform antigen retrieval by placing the sections in 10 mM sodium citrate buffer (pH 6.0) for 15 min. The sections were incubated with primary antibodies against β-catenin, α-sma, and CD44 (1:100, Abcam) overnight at 4°C. HRP-linked secondary antibody was used for incubation for 1 h at room temperature, and the nuclei were stained with hematoxylin.

### Hematoxylin–Eosin Staining

Mouse livers with metastatic tumor tissue were fixed in formaldehyde, embedded in paraffin, and cut into 4-μm-thick sections. The sections were then dewaxed with xylene and hydrated with gradient ethanol before staining with hematoxylin for 5 min and soaking in tap water for 5 min. The sections were subsequently stained with eosin solution for 2 min before conventional dehydration, vitrification and sealing with neutral resin. A Nikon imaging system (DS-U3, Tokyo, Japan) was used to obtain photos.

### Preparation of YJHJD

YJHJD comprises the 10 types of Chinese herbal medicines listed in **Table 2**. All herbs were obtained from the Affiliated Hospital of Nanjing University of Chinese Medicine and Jiangsu Province Hospital of TCM. YJHJD granules were dissolved in 165 ml of double-steamed water, heated, and stirred until boiling for 20 min. The liquid was concentrated to ensure the crude drug concentrations of 1.7–3.4 g/ml. The herb solution was then centrifuged at 10,000 *g*/min for 5 min and the supernatant was collected and subpackaged. The YJHJD drug serum used for the *in vitro* experiments was prepared according to the equivalent dose conversion formula (17). Pathogen-free Sprague–Dawley rats were administered 2 ml of YJHJD solution by gavage twice daily for 14 days (0.85 and 3.4 g/ml, respectively, in the YJHJ low-dose [DL] and YJHJ high-dose [DH] groups). Arterial blood was collected 1 h after the last drug administration and centrifuged at 3,000 r/min. The supernatants were stored at −80°C after inactivation and filtration sterilization.

**Table 2:** Yiqi Jianpi Huayu Jiedu decoction (YJHJD) composition.

Chinese name	Latin name	Doses (g)
Huang Qi	Astragalus Membranaceus	15
Dang Shen	Codonopsis Pilosula	15
Bai Zhu	Rhizomes Atractylodis Macrocephalae	10
Fu Ling	Poria Cocos	15
Shan Yao	Chinese Yam	15
Wu Mei	Smoked Plum	10
Cu San Leng	Vinegar Rhizoma Sparganii	10
Cu E Zhu	Vinegar Curcuma Zedoary	10
Xian He Cao	Herba Agrimoniae	30
Bai Jiang Cao	Defeat Sauce	30
Gan Cao	Radix Glycyrrhizae	5

### High-Performance Liquid Chromatography Analysis of YJHJD

A total of 100 µl of YJHJD at a concentration of 2 g/ml was diluted in methanol and then mixed. The solution was then centrifuged at 12,000 r/min for 10 min and the supernatant was collected. Separation was performed on an Agilent SB-C 18 column (100 mm × 4.60 mm, 1.8 µm). The mobile phase consisted of methyl alcohol (A), acetonitrile (B), and 0.1% formic acid. Gradient elution was performed according to the protocol. The temperature of the column was maintained at 40°C, with a sample volume of 10 µl.

### *In Vivo* Liver Metastasis Assay

A total of 30 BALB/c mice (6–8 weeks of age, 15 male and 15 female) were obtained from Beijing Vital River Laboratory Animal Technology Co., Ltd. The mice were anesthetized by intraperitoneal injection of 1% pentobarbital sodium. A total of 10 μl of CT26-GFP cell suspension (5 × 10^7^/ml) was injected under the spleen envelope in each. The mice were randomly divided into three groups (model, YJHJDL, and YJHJDH) on the third day after surgery. Mice in the YJHJDL (1.7 g/ml, 0.2 ml/20 g) and YJHJDH (3.4 g/ml, 0.2 ml/20 g) groups were administered YJHJD by gavage twice daily, while the mice in the model group were administered the same amount of normal saline for 21 days. The mice were sacrificed 2 h after the last drug administration. The livers were removed for photography and immediately frozen in liquid nitrogen for further analysis.

### Statistical Analysis

The results are presented as means ± SD. Statistical analysis was conducted using IBM SPSS Statistics for Windows, version 23.0 (IBM Corp., Armonk, NY, USA) with one-way ANOVA, followed by Duncan’s test. Statistical significance was defined as *p* < 0.05 or 0.01.

## Results

### Hsa-miR-374a-3p Is Downregulated in the Plasma of Patients With Hepatic Metastasis of COAD and Is Correlated to Poorer Prognosis

**Figure 1:**
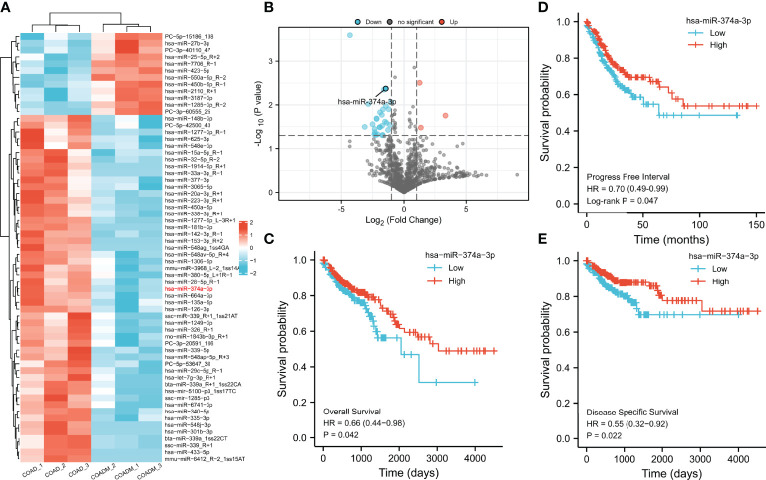
Decreased expression of hsa-miR-374a-3p is bound up with poor prognosis of COAD patients. **(A, B)** Plasma from 6 COAD patients was collected for analysis of microRNA expression profiles by high-throughput RNA-seq. **(C–E)** Data from TCGA was used to verify the association between expression of has-miR-374a-3p and prognosis (PFS, OS, and DSS) of COAD.

Plasma from a cohort of six patients diagnosed with COAD (three with hepatic metastasis [COAD_M] and three without) was analyzed to identify expression profiles using miRNA high-throughput RNA-seq analysis. The results showed that compared to the COAD group, the levels of 12 miRNAs increased and 51 miRNAs decreased in patients with COAD_M (**Figures 1A, B**). Subsequently, the Kaplan–Meier plotter was used to analyze the association between these miRNAs and clinical prognosis. Among the eight paracancerous tissues and 457 COAD samples from the TCGA database, low hsa-miR-374a-3p expression in tumor tissues was significantly correlated with worse overall survival (OS) (*p* = 0.042), disease-specific survival (DSS) (*p* = 0.022), and progression-free interval (PFI) (*p* = 0.047) (**Figures 1C–E**).

### Decreased Hsa-miR-374a-3p Is Related to the Malignant Progression of COAD

To investigate the clinical significance of hsa-miR-374a-3p, 66 patients were divided into high- and low-expression groups based on the mean plasma hsa-miR-374a-3p expression. The association between clinical or pathological features and hsa-miR-374a-3p expression was further analyzed. The data showed that low expression of miR-374a-3p in the plasma of patients with COAD was significantly related to tumor size (*p* = 0.013), T stage (*p* = 0.012), and lymphatic metastasis (*p* = 0.002), but not to patient age (*p* = 0.323), sex (*p* = 0.453), and tumor location (*p* = 0.447) (**Table 3**).

**Table 3:** Relationship between clinical or pathological characteristics and hsa-miR-374a-3p levels.

Variable	hsa-miR-374a-3p			
	*N* (%)	High expression (*n* = 33)	Low expression (*n* = 33)	*p*-value
**Age (years)**				0.323
≥60	36 (54.5)	20	16	
<60	30 (45.5)	13	17	
**Sex**				0.453
Male	39 (59.1)	18	21	
Female	27 (40.9)	15	12	
**Location**				0.447
Left	25 (37.9)	14	11	
Right	41 (62.1)	19	22	
**Tumor size** **(cm)**				0.013*
≥5	38 (57.6)	14	24	
<5	28 (42.4)	19	9	
**T stage**				0.012*
T 1–2	26 (39.4)	18	8	
T 3–4	40 (60.6)	15	25	
**N stage**				0.002*
N 0	17 (25.8)	14	3	
N 1–2	49 (74.2)	19	30	

*P < 0.05.

### WNT3 Is Potentially Targeted by and Expresses Inversely to Hsa-miR-374a-3p

**Figure 2:**
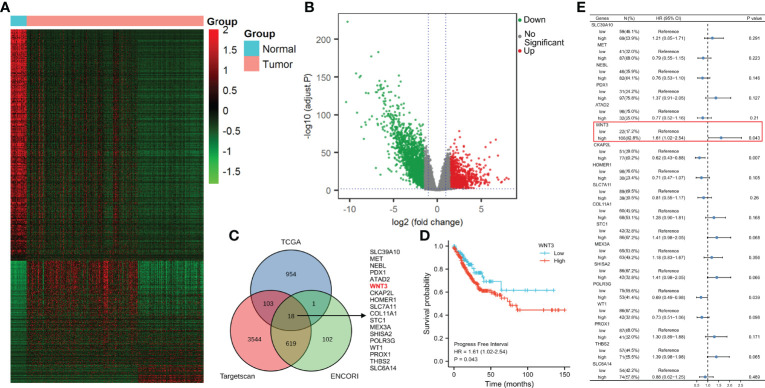
WNT3 is upregulated in COAD tissue and is a predictor of poor prognosis of COAD. **(A, B)** Analysis of mRNA expression profiles was performed through data from TCGA. **(C)** Potential target genes of hsa-miR-374a-3p were predicted *via* taking the intersection of TCGA, TargetScan Human 7.2, and ENCORI. **(D)** Prognostic function of WNT3 in COAD was explored by analyzing data of TCGA. **(E)** PFI of 18 genes was presented by a forest plot.

Differences in mRNA expression were analyzed using COAD data from TCGA. We discovered 1,078 upregulated and 2,048 downregulated genes in colorectal cancer (CRC) compared to paracancerous tissue (**Figures 2A, B**). Next, we predicted 4,284 and 740 potential target genes of hsa-miR-374a-3p using two public miRNA prediction databases (TargetScan Human 7.2 and ENCORI). Eighteen genes were initially screened based on their identification in the differential expression analysis from TCGA (upregulated) and the miRNA prediction databases (**Figure 2C**). Furthermore, prognostic analysis of 41 paracancerous tissues and 480 COAD samples from TCGA was conducted using the Kaplan–Meier plotter. WNT3 was finally identified as it was the only gene whose high expression was associated with poorer patient PFI (*p* = 0.043) (**Figures 2D, E**).

**Figure 3:**
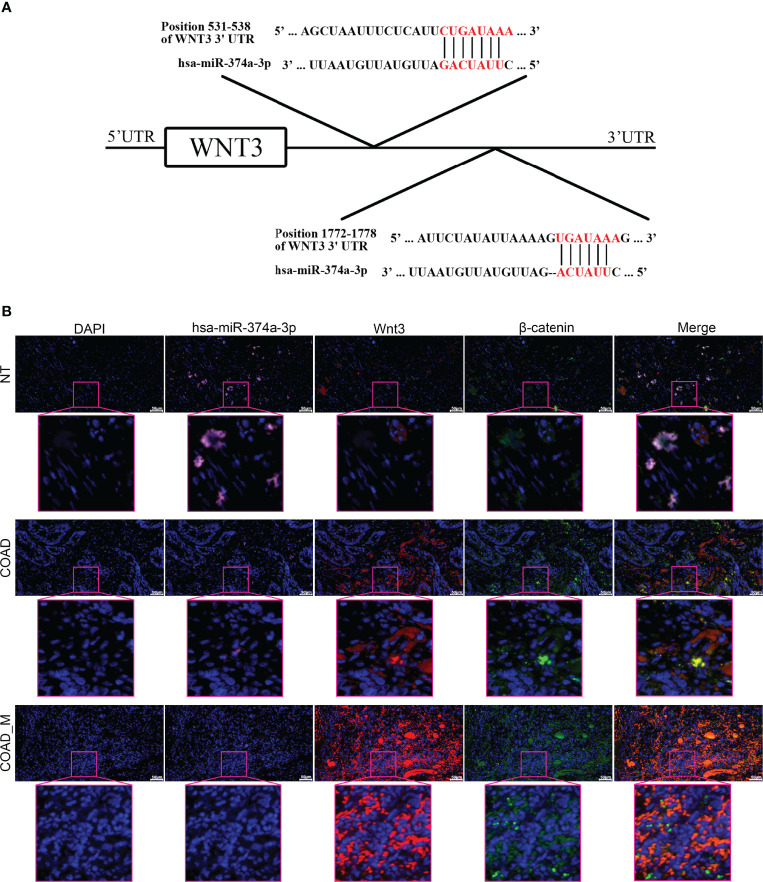
WNT3 is potentially targeted by hsa-miR-374a-3p and expression of WNT3 is negatively linked to hsa-miR-374a-3p in COAD. **(A)** Feasible binding sites were predicted using the TargetScan Human 7.2 database. **(B)** Expression of hsa-miR-374a-3p, Wnt3, and β-catenin in COAD specimens was probed *via* FISH and immunofluorescence experiments.

The TargetScan Human 7.2 database showed that hsa-miR-374a-3p can pair with positions 531–538 and 1772–1778 of the WNT3 mRNA 3’-UTR (**Figure 3A**). In addition, three groups of samples containing tumor-adjacent, COAD, and hepatic metastasis tissues from three patients who underwent surgery were obtained for further exploration. The results showed that hsa-miR-374a-3p was downregulated gradually in the tumor-adjacent, COAD, and hepatic metastasis tissues, while the expression of WNT3 and its downstream molecule β-catenin showed the opposite trend (**Figure 3B**). Thus, *WNT3* is potentially targeted by and has an adverse relationship with hsa-miR-374a-3p in COAD.

### Hsa-miR-374a-3p/WNT3 Axis-Mediated β-Catenin Signaling Regulates EMT and Cellular Plasticity in COAD Cells

**Figure 4:**
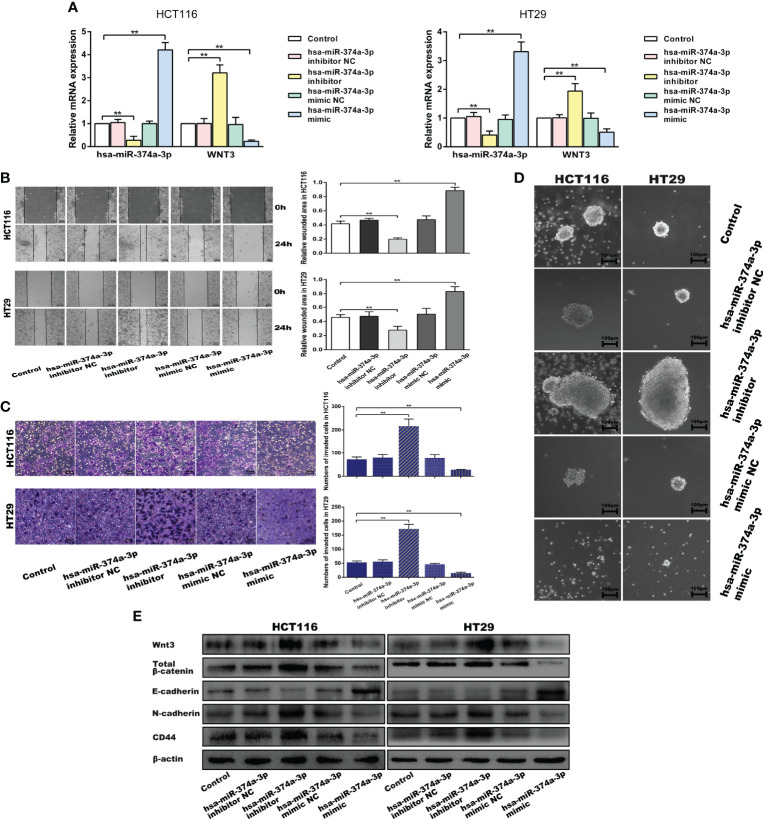
Hsa-miR-374a-3p plays an inhibitive role in the motility and cellular plasticity of COAD cells by suppressing WNT3/β-catenin signaling. **(A)** mRNA expression of hsa-miR-374a-3p and WNT3 was detected by PT-PCR assay. **(B)** Wound-healing assay was performed to assess the migration ability of CAOD cells. **(C)** Transwell assay was used to evaluate the invasion ability. **(D)** Spheroid formation assay proceeded to estimate the cellular plasticity. **(E)** Protein levels of WNT3/β-catenin, EMT, and cellular plasticity-related molecules were tested by Western blot experiment. *P < 0.05, **P < 0.01 compared to control group.

**Figure 5:**
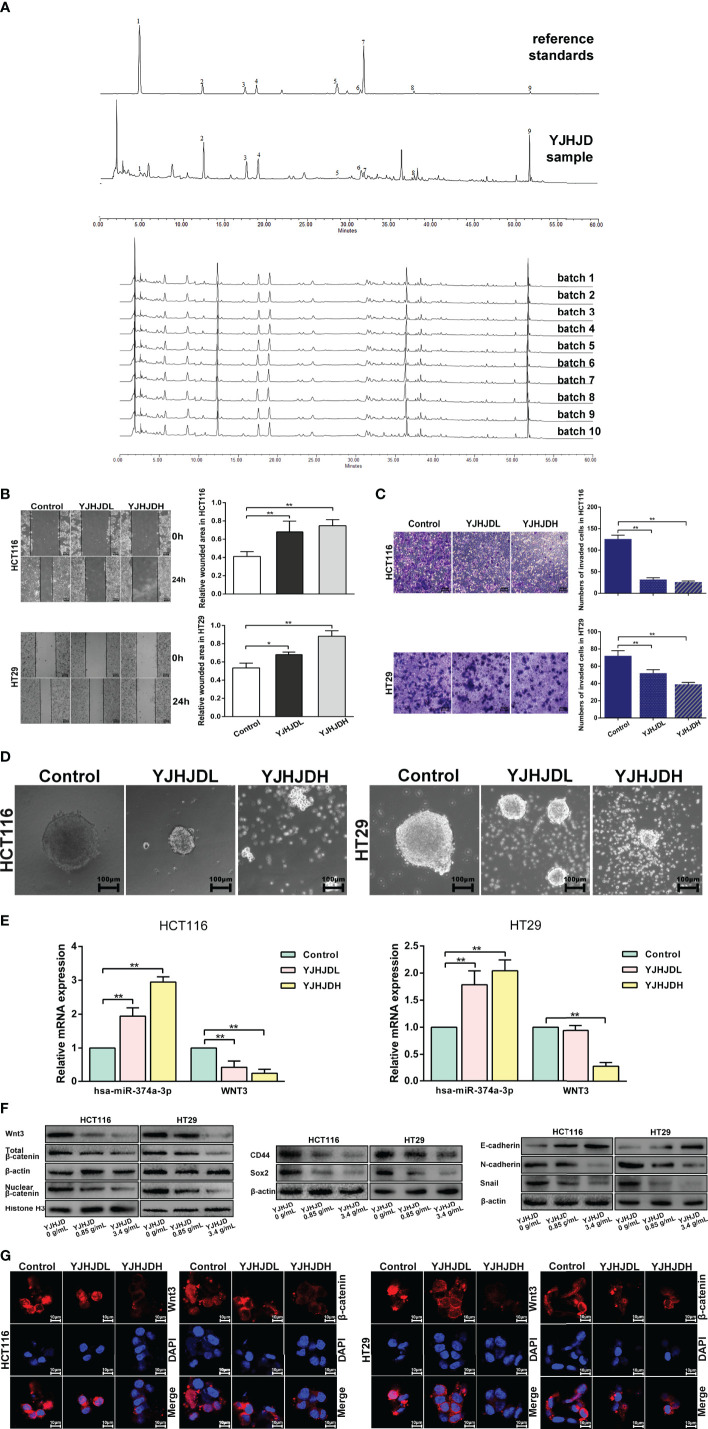
YJHJD prohibits EMT, cellular plasticity, and hsa-miR-374a-3p/WNT3/β-catenin axis-related molecules in COAD cells. **(A)** The HPLC fingerprint of YJHJD was established and compared with the reference standards. **(B)** Migration ability was tested by wound-healing assay. **(C)** Cell invasion ability was measured through transwell assay. **(D)** Cellular plasticity was assessed by spheroid formation assay. **(E)** mRNA expression of hsa-miR-374a-3p and WNT3 in response to YJHJD was evaluated by PT-PCR assay. **(F)** WNT3/β-catenin, EMT, and cellular plasticity-associated markers at protein levels were estimated by Western blot assay. **(G)** Protein expression and location of Wnt3 and β-catenin in COAD cells were tested *via* immunofluorescence analysis. *P < 0.05, **P < 0.01 compared to control group.

We performed wound-healing, transwell, and spheroid formation assays to verify that hsa-miR-374a-3p silencing increased cell motility and colony formation, while hsa-miR-374a-3p overexpression inhibited these effects in COAD cells (**Figures 4B–D**). COAD cells showed downregulated E-cadherin and upregulated N-cadherin and CD44 in response to hsa-miR-374a-3p silencing, while overexpression of hsa-miR-374a-3p led to adverse variations in related molecules (**Figure 5E**). Moreover, Wnt3 was upregulated when hsa-miR-374a-3p was silenced but downregulated when hsa-miR-374a-3p was overexpressed at both the mRNA and protein levels. β-catenin protein expression showed a similar trend in HCT116 and HT29 cells (**Figures 4A, E**).

### The Chemical Components of YJHJD

The HPLC fingerprint of the YJHJD decoction was compared to reference standards (**Figure 5A**). Gallic acid, neochlorogenic acid, chlorogenic acid, cryptochlorogenic acid, ferulic acid, licorice glycosides, calycosin-7-glucoside, Dangshen acetylene glycosides, and glycyrrhizic acid were detected, several of which exhibited anticancer activity. Gallic acid induced apoptosis, suppressed proliferation, and decreased cell viability (18, 19). Chlorogenic acid induced reactive oxygen species generation, cell cycle arrest, and apoptosis (20, 21). Ferulic acid induced apoptosis (22). Glycyrrhizic acid treatment decreased cell viability, motility, and cloning ability and increased apoptosis (23). From the literature review, peak 1 (gallic acid) might be derived from *Angelica sinensis* or *Oldenlandia diffusa*; peak 2 (neochlorogenic acid), peak 3 (chlorogenic acid), and peak 4 (cryptochlorogenic acid) might be derived from *Angelica sinensis*; peak 5 (ferulic acid) might be derived from *Angelica sinensis, Rhizoma atractylodis macrocephalae, Rhizoma sparganii*, or *Curcuma zedoary*; peak 6 (licorice glycosides) and peak 9 (glycyrrhizic acid) might be derived from licorice; peak 7 (calycosin-7-glucoside) might be derived from *Astragalus membranaceus*; and peak 8 (Dangshen acetylene glycosides) might be derived from *Codonopsis pilosula*.

### YJHJD Inhibits EMT and Colony Formation Ability of COAD cells, and Regulates the Expression of Hsa-miR-374a-3p and WNT3

The migration assay showed a relatively increased wound area, suggesting that YJHJD effectively inhibited the migration ability of HCT116 and HT29 cells (**Figure 5B**). The transwell assay showed a gradual reduction in the number of invading cells, confirming that YJHJD decreased the invasion ability of both types of colon cancer cells (**Figure 5C**). The spheroid formation assay demonstrated that YJHJD inhibited colon cancer cell colony formation in a dose-dependent manner (**Figure 5D**).

The results of Western blotting showed that YJHJD upregulated E-cadherin and downregulated N-cadherin, Snail, CD44, and Sox2 in a dose-dependent manner (**Figure 5F**). Additionally, RT-PCR and Western blot assays showed increased hsa-miR-374a-3p and decreased WNT3 response to YJHJD (**Figure 5E**). In addition, YJHJD significantly reduced cytoplasmic and nuclear β-catenin protein expression in both HCT116 and HT29 cells (**Figure 5F**). The reduced Wnt3 and β-catenin signals were further explored by immunofluorescence (**Figure 5G**).

### YJHJD Suppresses EMT and Cellular Plasticity Through Hsa-miR-374a-3p/WNT3 Axis-Mediated β-Catenin Signaling

**Figure 6:**
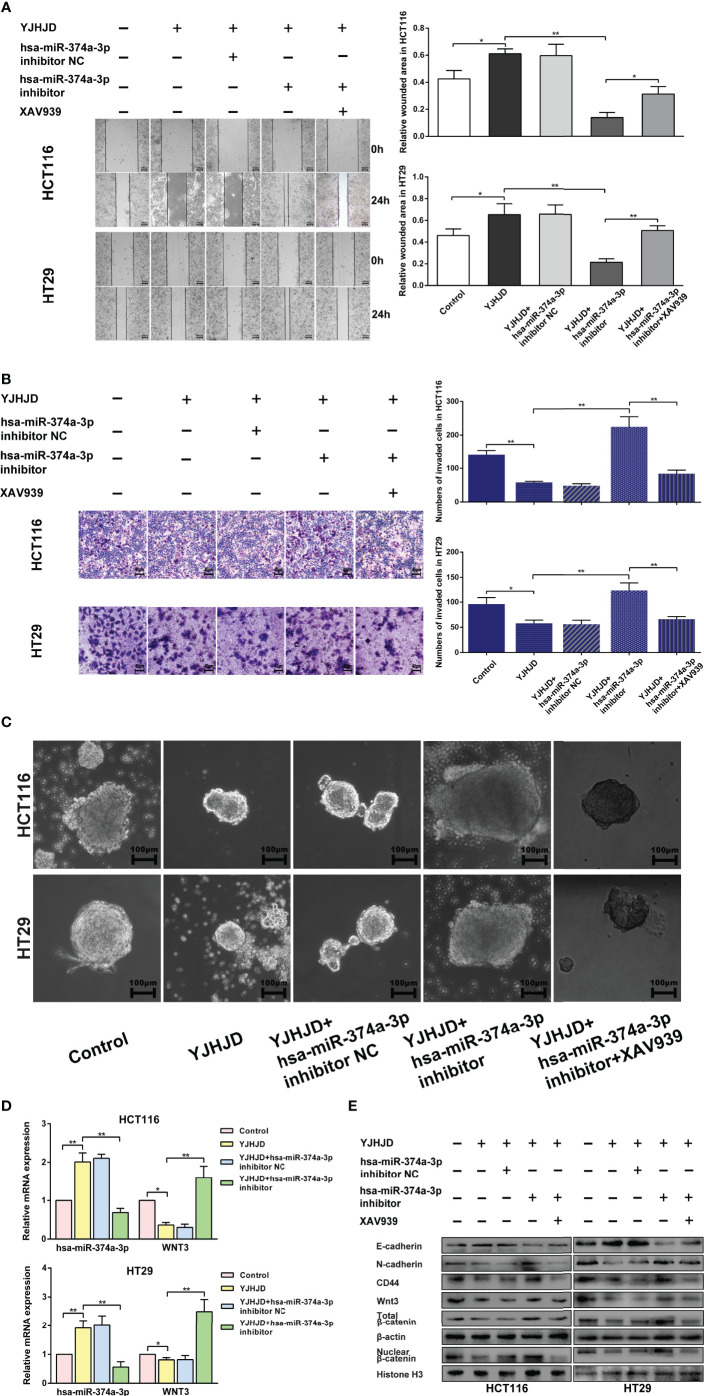
The hsa-miR-374a-3p/WNT3/β-catenin axis plays a vital role in the suppressive effect of YJHJD. **(A)** A wound-healing experiment was performed to test cell migration ability. **(B)** Transwell assay was performed to explore cell invasion ability. **(C)** Spheroid formation assay was carried out to appraise cellular plasticity. **(D)** Hsa-miR-374a-3p and WNT3 mRNA expression was detected by PT-PCR experiment. **(E)** WNT3/β-catenin, EMT, and cellular plasticity-related protein expression was researched through Western blot assay. *P < 0.05, **P < 0.01 compared to control group. *P < 0.05, **P < 0.01 compared to control group.

HCT116 and HT29 cells with hsa-miR-374a-3p silencing showed decreased hsa-miR-374a-3p and increased WNT3 mRNA expression, in contrast to cells without silencing exposed to YJHD (**Figure 6D**). Silencing of hsa-miR-374a-3p also counteracted the inhibitory effect of YJHJD on cell motility and clonality (**Figures 6A–C**), as well as β-catenin, EMT, and cellular plasticity-related molecular changes at the protein level (**Figure 6E**). In addition, the application of the specific β-catenin inhibitor XAV939 partially restored the suppressive effect of YJHJD, which was weakened by transfection silencing of hsa-miR-374a-3p in colon cancer cells (**Figure 6**).

### YJHJD Represses Hepatic Metastasis of Colon Cancer by Inhibiting Hsa-miR-374a-3p/WNT3 Axis-Regulated EMT and Cellular Plasticity

**Figure 7:**
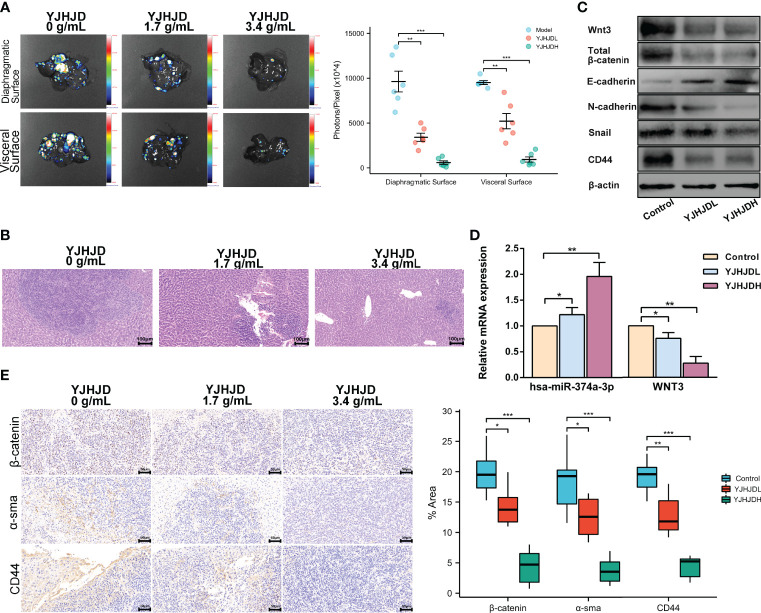
YJHJD restrains liver metastasis of COAD *via* reversing hsa-miR-374a-3p/WNT3/β-catenin-mediated EMT and cellular plasticity. **(A)** Fluorescence intensity on behalf of liver metastases was assessed by the Live Animal Analysis System. **(B)** Liver metastatic lesions and tumor infiltration was evaluated by HE staining. **(C)** mRNA levels of hsa-miR-374a-3p, WNT3 was tested by PT-PCR assay. **(D)** Protein expression of WNT3/β-catenin, EMT, and cellular plasticity relevant molecules was studied through Western blot assay. **(E)** Expression of β-catenin and α-sma in the liver metastatic tumor tissues was examined *via* IHC analysis. *P <0.05, **P < 0.01, *** P < 0.001 compared to control group.

A hepatic metastasis model was established by injecting CT26 cells into the spleen of mice to investigate the effect of YJHJD on colon cancer cell invasion *in vivo*. The groups treated with YJHJD showed significantly reduced liver metastases, especially in the high-dose group (**Figure 7A**). HE staining verified the remarkably decreased number and diameter of hepatic metastatic nodules in mice in the YJHJD group as well (**Figure 7B**). The expression of related molecules in liver metastatic tumor tissues was also investigated. RT-PCR assay showed that YJHJD effectively upregulated hsa-miR-374a-3p and downregulated WNT3 at the mRNA level (**Figure 7D**). Western blotting confirmed that YJHJD upregulated the protein expression of E-cadherin and downregulated the expression of Wnt3, β-catenin, N-cadherin, Snail, CD44, and Sox2 (**Figure 7C**). Meanwhile, immunohistochemical staining confirmed the decreased expression of β-catenin, α-sma, and CD44 in the liver metastases of colon cancer (**Figure 7E**).

## Discussion

Our previous clinical research validated that the Chinese herbal formulation based on the method of “nourish qi, invigorate the spleen, remove blood stasis and detoxify” prolonged disease-free survival (DFS), reduced the risk of recurrence and metastasis, and improved the quality of life of patients with stage II or III gastric cancer after radical gastrectomy (24). In addition, this therapy increased the survival rate and inhibited CRC liver metastasis in a nude mouse model by activating the innate immune system (25). This treatment method also inhibited COAD cell proliferation by inducing G0/G1-phase cell cycle arrest and cell apoptosis (26). The present study further explored the mechanism underlying the inhibitory effects of YJHJD on COAD.

MiRNAs play a vital role in the biological behavior of CRC, as well as in patient prognosis. Hsa-miR-374a is correlated with CRC development and prognostic indicators and can significantly reduce the mortality risk despite the tumor site, as high hsa-miR-374a expression is associated with better survival (27, 28). After surgical resection of colorectal advanced adenomas (CAA) or CRC, patients showed upregulated plasma levels of miR-374a (29). We observed that hsa-miR-374a-3p was downregulated in the plasma of patients diagnosed with COAD with liver metastasis compared to those without. Moreover, decreased levels of hsa-miR-374a-3p in COAD tissues were associated with reduced OS, DSS, and PFI. Our retrospective study further indicated that patients with lower plasma expression of miR-374a-3p might have a larger tumor size and more advanced T or N stages of COAD. The sequence of *in vitro* assays showed that hsa-miR-374a-3p silencing enhanced COAD cell migration, invasion ability, and cellular plasticity, whereas hsa-miR-374a-3p overexpression led to the opposite phenotype. Thus, we inferred that hsa-miR-374a-3p may serve as a tumor suppressor that inhibits COAD development and metastasis.

In the canonical Wnt pathway, Wnt ligands bind to the corresponding receptors, leading to the nuclear translocation of β-catenin and activation of target genes (30). Wnt/β-catenin signaling is highly activated in several malignancies, including colon cancer (31). Hyperactivated Wnt/β-catenin signaling is thought to promote CRC progression by regulating the EMT, as overexpression of nuclear β-catenin and continuous activation of Wnt/β-catenin signaling have been reported in aggressive colon cancer (32). The Wnt/β-catenin pathway also plays a major role in maintaining cellular plasticity in colon cancers (33). Moreover, the reciprocal action between microRNAs and the canonical Wnt pathway is a key regulator and prognostic factor in colon tumors (34). Our analysis of COAD data from TCGA revealed 1,078 upregulated genes, among which the expression of WNT3 was inversely connected with PFI. TargetScan Human 7.2. indicated that the binding domain of hsa-miR-374a-3p might be located at positions 531–538 and 1772–1778 of the WNT3 3’-UTR. The expression of hsa-miR-374a-3p decreased gradually in tumor-adjacent, COAD, and hepatic metastasis tissues, while WNT3 and β-catenin showed opposite trends. In addition, silencing of hsa-miR-374a-3p induced the EMT and cellular plasticity of COAD cells, as well as the expression of Wnt3 and β-catenin, while overexpression of hsa-miR-374a-3p resulted in a suppressive effect. Therefore, we concluded that hsa-miR-374a-3p regulated WNT3 expression and further mediated β-catenin signaling to inhibit EMT and cellular plasticity in COAD.

Traditional Chinese medicine is an ancient practice of medicine with a long-standing history that has been applied in clinics for thousands of years and has potential advantages in anti-tumor efficiency (35, 36). The YJHJD decoction is a traditional Chinese medicine compound based on the theory of “spleen deficiency and stasis toxin” that has been applied clinically for years. First, we demonstrated that YJHJD reduced COAD cell migration, invasion, and colony formation potential in a dose-dependent manner. Additionally, YJHJD reversed the EMT and the expression of cellular plasticity-related molecules, with increased hsa-miR-374a-3p and Wnt3 and β-catenin inhibition. Furthermore, the suppressive role of YJHJD was neutralized when hsa-miR-374a-3p was silenced by transfection, whereas the application of the β-catenin inhibitor XAV939 partially restored the effect of YJHJD. Further *in vivo* experiments confirmed that YJHJD inhibited liver metastasis in a dose-dependent manner by upregulating hsa-miR-374a-3p, downregulating the Wnt3/β-catenin axis, and inhibiting the EMT and cellular plasticity of COAD.

**Figure 8:**
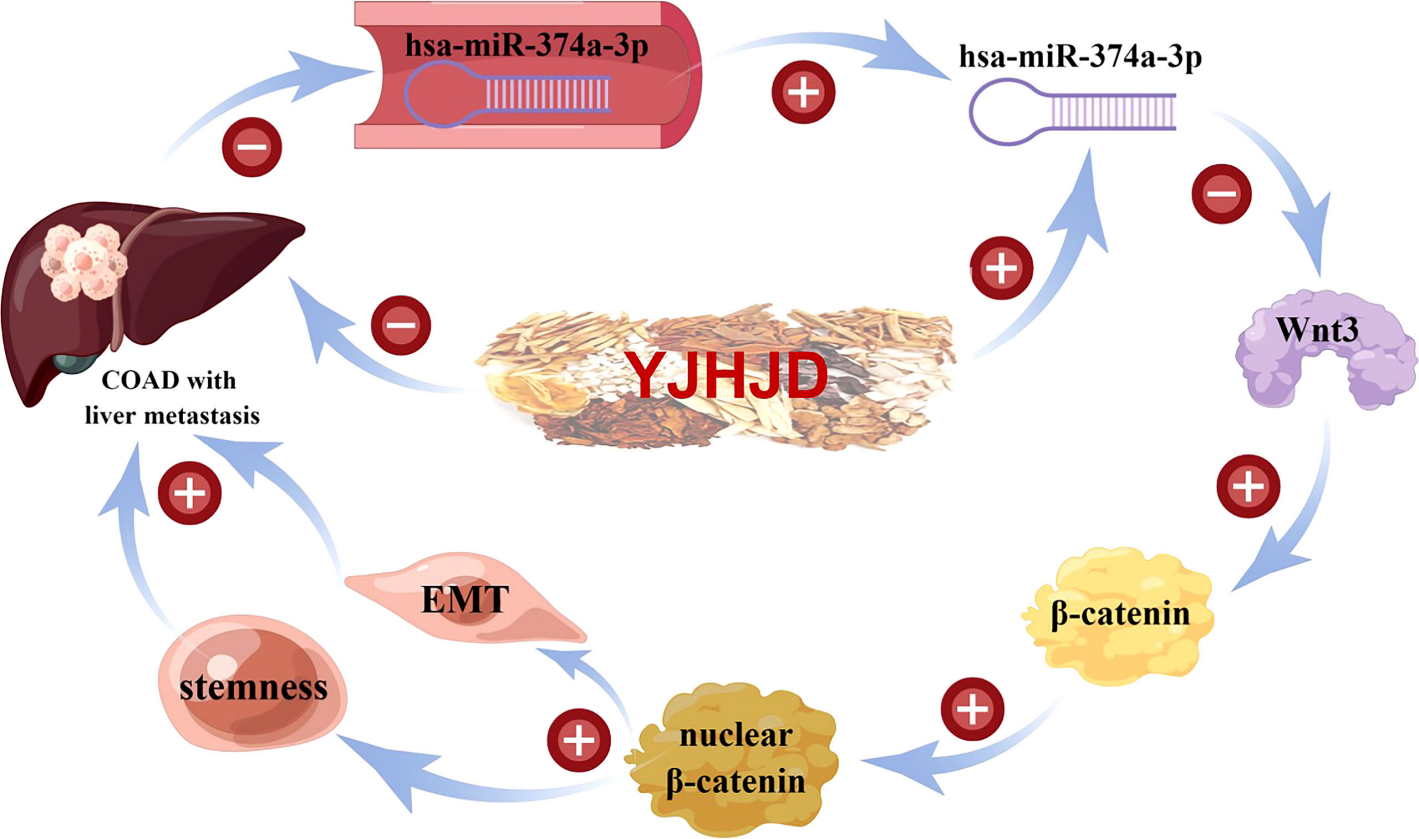
Mechanism flowchart of the effect of YJHJD on COAD.

Therefore, hsa-miR-374a-3p is a potential tumor-inhibiting gene in COAD and is closely related to patient prognosis. Notably, hsa-miR-374a-3p/Wnt3/β-catenin signal transduction played a suppressive role by regulating the EMT and cellular plasticity in COAD. The YJHJD decoction established in the theory of “spleen deficiency and stasis toxin” can be applied for the treatment of liver metastasis of COAD. The underlying mechanism is that YJHJD inhibits the EMT and cellular plasticity of COAD in an hsa-miR-374a-3p/Wnt3/β-catenin dependent manner (**Figure 8**). However, additional studies are needed to elucidate the specific mechanism by which YJHJD acts on hsa-miR-374a-3p and identify potential intermediate effector molecules between the hsa-miR-374a-3p/Wnt3/β-catenin axis and EMT or cellular plasticity.

## Data Availability Statement

The datasets presented in this study can be found in online repositories. The names of the repository/repositories and accession number(s) can be found at: https://www.ncbi.nlm.nih.gov/geo/query/acc.cgi?acc=GSE184669.

## Ethics Statement

The studies involving human participants were reviewed and approved by the Ethics Committee of Nanjing University of Chinese Medicine. The patients/participants provided their written informed consent to participate in this study. The animal study was reviewed and approved by the Ethics Committee of Nanjing University of Chinese Medicine.

## Author Contributions

Conceptualization: YZ, JZ, SL, and CW. Data management: JZ and CW. Formal analysis: QW, JQ, XZ, and HP. Methodology: TX and ZJ. Resources: CW. Writing—original draft: YZ and CW. Writing—review and editing: JZ and SL. All authors contributed to the article and approved the submitted version.

## Funding

This study was supported by the National Natural Science Foundation of China (82104950), the National Administration of Traditional Chinese Medicine: 2019 Project of building evidence based practice capacity for TCM (No.2019XZZX-ZL003), the National Traditional Chinese Medicine Inheritance and Innovation Platform Construction Project by National Administration of Traditional Chinese Medicine, Natural Science Foundation of Jiangsu Province (BK20191086, BK20201499), the Project of National Clinical Research Base of Traditional Chinese Medicine in Jiangsu Province, China (JD2019SZXYB01), the Medical Scientific Research Project of Jiangsu Health Commission (H2019094), and the College Project of Jiangsu Province Hospital of Chinese Medicine (Y2020CX57, Y2020CX67, and Y2021CX06).

## Conflict of Interest

The authors declare that the research was conducted in the absence of any commercial or financial relationships that could be construed as a potential conflict of interest.

## Publisher’s Note

All claims expressed in this article are solely those of the authors and do not necessarily represent those of their affiliated organizations, or those of the publisher, the editors and the reviewers. Any product that may be evaluated in this article, or claim that may be made by its manufacturer, is not guaranteed or endorsed by the publisher.

## References

[1] Sung H Ferlay J Siegel RL Laversanne M Soerjomataram I Jemal A. Global Cancer Statistics 2020: GLOBOCAN Estimates of Incidence and Mortality Worldwide for 36 Cancers in 185 Countries. CA Cancer J Clin (2021) 71(3):209–49. doi: 10.3322/caac.21660 33538338

[2] Cao W Chen HD Yu YW Li N Chen WQ. Changing Profiles of Cancer Burden Worldwide and in China: A Secondary Analysis of the Global Cancer Statistics 2020. Chin Med J (Engl) (2021) 134(7):783–91. doi: 10.1097/CM9.0000000000001474 PMC8104205 33734139

[3] Siegel RL Miller KD Fedewa SA Ahnen DJ Meester R Barzi A. Colorectal Cancer Statistic. CA Cancer J Clin (2017) 67(3):177–93. doi: 10.3322/caac.21395 28248415

[4] Zhu J Lian P Liu F Xu Y Xu J Guan Z. Phase II Trial of First-Line Chemoradiotherapy With Intensity-Modulated Radiation Therapy Followed by Chemotherapy for Synchronous Unresectable Distant Metastases Rectal Adenocarcinoma. Radiat Oncol (2013) 8:10. doi: 10.1186/1748-717X-8-10 23295152 PMC3552710

[5] Stemmler MP Eccles RL Brabletz S Brabletz T. Non-Redundant Functions of EMT Transcription Factors. Nat Cell Biol (2019) 21(1):102–12. doi: 10.1038/s41556-018-0196-y 30602760

[6] Bakir B Chiarella AM Pitarresi JR Rustgi AK. EMT, MET, Plasticity, and Tumor Metastasis. Trends. Cell Biol (2020) 30(10):764–76. doi: 10.1016/j.tcb.2020.07.003 PMC7647095 32800658

[7] Soundararajan R Paranjape AN Maity S Aparicio A Mani SA. EMT, Stemness and Tumor Plasticity in Aggressive Variant Neuroendocrine Prostate Cancers. Biochim Biophys Acta Rev Cancer (2018) 1870 (2):229–38. doi: 10.1016/j.bbcan.2018.06.006 PMC6496942 29981816

[8] Botchkina G. Colon Cancer Stem Cells–From Basic to Clinical Application. Cancer Lett (2013) 338(1):127–40. doi: 10.1016/j.canlet.2012.04.006 22537805

[9] Lin S Gregory RI. MicroRNA Biogenesis Pathways in Cancer. Nat Rev Cancer (2015) 15(6):321–33. doi: 10.1038/nrc3932 PMC4859809 25998712

[10] Zhen Y Fang W Zhao M Luo R Liu Y Fu Q. miR-374a-CCND1-Ppi3k/AKT-C-JUN Feedback Loop Modulated by PDCD4 Suppresses Cell Growth, Metastasis, and Sensitizes Nasopharyngeal Carcinoma to Cisplatin. Oncogene (2017) 36(2):275–85. doi: 10.1038/onc.2016.201 27270423

[11] Pan Z Shi Z Wei H Sun F Song J Huang Y. Magnetofection Based on Superparamagnetic Iron Oxide Nanoparticles Weakens Glioma Stem Cell Proliferation and Invasion by Mediating High Expression of MicroRNA-374a. J Cancer (2016) 7(11):1487–96. doi: 10.7150/jca.15515 PMC4964133 27471565

[12] Cai J Guan H Fang L Yang Y Zhu X Yuan J. MicroRNA-374a Activates Wnt/β-Catenin Signaling to Promote Breast Cancer Metastasis. J Clin Invest (2013) 123(2):566–79. doi: 10.1172/JCI65871 PMC3561816 23321667

[13] Kim JG Mahmud S Min JK Lee YB Kim H Kang DC. RhoA GTPase Phosphorylated at Tyrosine 42 by Src Kinase Binds to β-Catenin and Contributes Transcriptional Regulation of Vimentin Upon Wnt3A. Redox Biol (2021) 40:101842. doi: 10.1016/j.redox.2020.101842 33388549 PMC7788234

[14] Kozomara A Birgaoanu M Griffiths-Jones S. Mirbase: From microRNA Sequences to Function. Nucleic Acids Res (2019) 47(D1):D155–62. doi: 10.1093/nar/gky1141 PMC6323917 30423142

[15] de Planell-Saguer M Rodicio MC Mourelatos Z. Rapid *in Situ* Codetection of Noncoding RNAs and Proteins in Cells and Formalin-Fixed Paraffin-Embedded Tissue Sections Without Protease Treatment. Nat Protoc (2010) 5(6):1061–73. doi: 10.1038/nprot.2010.62 20539282

[16] Su D Guo X Huang L Ye H Li Z Lin L. Tumor-Neuroglia Interaction Promotes Pancreatic Cancer Metastasis. Theranostics. 10 (2020) 11):5029–47. doi: 10.7150/thno.42440 PMC7163446 32308766

[17] Wang Z Zhang Y Liu Q Sun L Lv M Yu P. Investigation of the Mechanisms of Genkwa Flos Hepatotoxicity by a Cell Metabolomics Strategy Combined With Serum Pharmacology in HL-7702 Liver Cells. *Xenobiotica* . 49 (2019) 2):216–26. doi: 10.1080/00498254.2018.1427905 29325475

[18] Subramanian AP Jaganathan SK Mandal M Supriyanto E Muhamad II. Gallic Acid Induced Apoptotic Events in HCT-15 Colon Cancer Cells. World. J Gastroenterol (2016) 22(15):3952–61. doi: 10.3748/wjg.v22.i15.3952 PMC4823245 27099438

[19] Forester SC Choy YY Waterhouse AL Oteiza PI. The Anthocyanin Metabolites Gallic Acid, 3-O-Methylgallic Acid, and 2,4,6-Trihydroxybenzaldehyde Decrease Human Colon Cancer Cell Viability by Regulating Pro-Oncogenic Signals. Mol Carcinog (2014) 53(6):432–9. doi: 10.1002/mc.21974 23124926

[20] Hou N Liu N Han J Yan Y Li J. Chlorogenic Acid Induces Reactive Oxygen Species Generation and Inhibits the Viability of Human Colon Cancer Cells. Anticancer Drugs (2017) 28(1):59–65. doi: 10.1097/CAD.0000000000000430 27603595

[21] Sadeghi ES Li XQ Ghorbani M Azadi B Kubow S. Chlorogenic Acid and Its Microbial Metabolites Exert Anti-Proliferative Effects, S-Phase Cell-Cycle Arrest and Apoptosis in Human Colon Cancer Caco-2 Cells. Int J Mol Sci (2018) 19(3):723. doi: 10.3390/ijms19030723 PMC5877584 29510500

[22] Senthil, Kumar C Thangam R Mary SA Kannan PR Arun G Madhan B. Targeted Delivery and Apoptosis Induction of Trans-Resveratrol-Ferulic Acid Loaded Chitosan Coated Folic Acid Conjugate Solid Lipid Nanoparticles in Colon Cancer Cells. Carbohydr Polym (2020) 231:115682. doi: 10.1016/j.carbpol.2019.115682 31888816

[23] Zuo Z He L Duan X Peng Z Han J. Glycyrrhizic Acid Exhibits Strong Anticancer Activity in Colorectal Cancer Cells *via* SIRT3 Inhibition. Bioengineered (2021) 13(2):2720–31. doi: 10.1080/21655979.2021.2001925 PMC8974138 34747319

[24] Shu P Tang H Zhou B Wang R Xu Y Shao J. Effect of Yiqi Huayu Jiedu Decoction on Stages II and III Gastric Cancer: A Multicenter, Prospective, Cohort Study. *Medicine (Baltimore)* 98 (2019) (47):e17875. doi: 10.1097/MD.0000000000017875 PMC6882584 31764782

[25] Zhou JY Chen M Wu CE Zhuang YW Chen YG Liu SL. The Modified Si-Jun-Zi Decoction Attenuates Colon Cancer Liver Metastasis by Increasing Macrophage Cells. BMC Complement Altern Med (2019) 19(1):86. doi: 10.1186/s12906-019-2498-4 31014289 PMC6477719

[26] Xi SY Teng YH Chen Y Li JP Zhang YY Liu SL. Jianpi Huayu Decoction Inhibits Proliferation in Human Colorectal Cancer Cells (SW480) by Inducing G0/G1-Phase Cell Cycle Arrest and Apoptosis. . Evid Based Complement Alternat Med (2015) 2015:236506. doi: 10.1155/2015/236506 26457107 PMC4589617

[27] Slattery ML Herrick JS Mullany LE Valeri N Stevens J Caan BJ. An Evaluation and Replication of miRNAs With Disease Stage and Colorectal Cancer-Specific Mortality. Int J Cancer (2015) 137(2):428–38. doi: 10.1002/ijc.29384 PMC4428989 25484364

[28] Slattery ML Pellatt AJ Lee FY Herrick JS Samowitz WS Stevens JR. Infrequently Expressed miRNAs Influence Survival After Diagnosis With Colorectal Cancer. Oncotarget (2017) 8(48):83845–59. doi: 10.18632/oncotarget.19863 PMC5663559 29137387

[29] O'Brien SJ Netz U Hallion J Bishop C Stephen V Burton J. Circulating Plasma microRNAs in Colorectal Neoplasia: A Pilot Study in Assessing Response to Therapy. Transl Oncol (2021) 14(1):100962. doi: 10.1016/j.tranon.2020.100962 33285367 PMC7720092

[30] Chizhikov VV Iskusnykh IY Steshina EY Fattakhov N Lindgren AG Shetty AS. Early Dorsomedial Tissue Interactions Regulate Gyrification of Distal Neocortex. Nat Commun (2019) 10(1):5192. doi: 10.1038/s41467-019-12913-z 31729356 PMC6858446

[31] Ji P Zhou Y Yang Y Wu J Zhou H Quan W. Myeloid Cell-Derived LL-37 Promotes Lung Cancer Growth by Activating Wnt/β-Catenin Signaling. Theranostics (2019) 9(8):2209–23. doi: 10.7150/thno.30726 PMC6531301 31149039

[32] Ahmad R Kumar B Chen Z Chen X Müller D Lele SM. Loss of Claudin-3 Expression Induces IL6/gp130/Stat3 Signaling to Promote Colon Cancer Malignancy by Hyperactivating Wnt/β-Catenin Signaling. Oncogene (2017) 36(47):6592–604. doi: 10.1038/onc.2017.259 PMC6512312 28783170

[33] Sacchetti A Teeuwssen M Verhagen M Joosten R Xu T Stabile R. Phenotypic Plasticity Underlies Local Invasion and Distant Metastasis in Colon Cancer. eLife (2021) 10:e61461. doi: 10.7554/eLife.61461 34036938 PMC8192123

[34] Uddin MN Li M Wang X. Identification of Transcriptional Markers and microRNA-mRNA Regulatory Networks in Colon Cancer by Integrative Analysis of mRNA and microRNA Expression Profiles in Colon Tumor Stroma. Cells (2019) 8(9):1054. doi: 10.3390/cells8091054 PMC6769865 31500382

[35] Qian Q Chen W Cao Y Cao Q Cui Y Li Y. Targeting Reactive Oxygen Species in Cancer *via* Chinese Herbal Medicine. Oxid Med Cell Longev (2019) 2019:9240426. doi: 10.1155/2019/9240426 31583051 PMC6754955

[36] Hao H. The Development of Online Doctor Reviews in China: An Analysis of the Largest Online Doctor Review Website in China. J Med Internet Res (2015) 17(6):e134. doi: 10.2196/jmir.4365 26032933 PMC4526894

